# Facial phenotypes in subgroups of prepubertal boys with autism spectrum disorders are correlated with clinical phenotypes

**DOI:** 10.1186/2040-2392-2-15

**Published:** 2011-10-14

**Authors:** Kristina Aldridge, Ian D George, Kimberly K Cole, Jordan R Austin, T Nicole Takahashi, Ye Duan, Judith H Miles

**Affiliations:** 1 Department of Pathology and Anatomical Sciences, University of Missouri School of Medicine, One Hospital Dr, M309 Med Sci Bldg, Columbia, MO 65212, USA; 2 Thompson Center for Autism and Neurodevelopmental Disorders, University of Missouri, 205 Portland St, Columbia, MO 65211, USA; 3 Department of Computer Science, University of Missouri, 209 Engineering Building West, Columbia, MO 65211, USA; 4 Department of Child Health, University of Missouri School of Medicine, One Hospital Dr, N712, Columbia, MO 65212, USA

**Keywords:** autism, neurodevelopment, anthropometry, facial phenotype, biomarker, craniofacial genetics

## Abstract

**Background:**

The brain develops in concert and in coordination with the developing facial tissues, with each influencing the development of the other and sharing genetic signaling pathways. Autism spectrum disorders (ASDs) result from alterations in the embryological brain, suggesting that the development of the faces of children with ASD may result in subtle facial differences compared to typically developing children. In this study, we tested two hypotheses. First, we asked whether children with ASD display a subtle but distinct facial phenotype compared to typically developing children. Second, we sought to determine whether there are subgroups of facial phenotypes within the population of children with ASD that denote biologically discrete subgroups.

**Methods:**

The 3dMD cranial System was used to acquire three-dimensional stereophotogrammetric images for our study sample of 8- to 12-year-old boys diagnosed with essential ASD (*n *= 65) and typically developing boys (*n *= 41) following approved Institutional Review Board protocols. Three-dimensional coordinates were recorded for 17 facial anthropometric landmarks using the 3dMD Patient software. Statistical comparisons of facial phenotypes were completed using Euclidean Distance Matrix Analysis and Principal Coordinates Analysis. Data representing clinical and behavioral traits were statistically compared among groups by using χ^2 ^tests, Fisher's exact tests, Kolmogorov-Smirnov tests and Student's *t*-tests where appropriate.

**Results:**

First, we found that there are significant differences in facial morphology in boys with ASD compared to typically developing boys. Second, we also found two subgroups of boys with ASD with facial morphology that differed from the majority of the boys with ASD and the typically developing boys. Furthermore, membership in each of these distinct subgroups was correlated with particular clinical and behavioral traits.

**Conclusions:**

Boys with ASD display a facial phenotype distinct from that of typically developing boys, which may reflect alterations in the prenatal development of the brain. Subgroups of boys with ASD defined by distinct facial morphologies correlated with clinical and behavioral traits, suggesting potentially different etiologies and genetic differences compared to the larger group of boys with ASD. Further investigations into genes involved in neurodevelopment and craniofacial development of these subgroups will help to elucidate the causes and significance of these subtle facial differences.

## Background

Autism is a spectrum of disorders (ASDs) united by a common *Diagnostic and Statistical Manual of Mental Disorders, Fourth Edition *(DSM-IV)-defined [1] behavioral phenotype. Research into this disorder is increasingly focused on both genetic causes and neuroanatomical bases for the behavioral phenotypes. Thus far attempts to discover major autism susceptibility genes have been largely unsuccessful, with approximately only 15% to 20% of cases of autism linked to specific genes, chromosomal aneuploidy or recognized syndromes [2-4]. The rest remain idiopathic. A primary reason for the lack of progress in understanding the etiology and genetic underpinnings of ASD is undoubtedly the significant heterogeneity within both behavioral and clinical phenotypes.

The neurodevelopmental model of ASD [5] suggests that changes in embryonic developmental patterns result in the spectrum of ASD phenotypes and that these changes may result from permutations of genetics, the environment, or the interaction of the two. This model posits that the brain is altered during embryonic development, a time when the brain is intimately tied to developing facial tissues via genetic signaling, biomechanical and biochemical mechanisms [6-13]. The face and brain form a vast but segmented population of cells whose differentiation and identity are established through intricate signaling mechanisms. It has been stated that the brain is the foundation on which the various parts of the developing face grow [14]. The phrase, "The face predicts the brain" [15], has been employed frequently to explain developmental disorders such as holoprosencephaly. Thus changes to the developing brain may be reflected in the face [15-17].

The face develops from populations of neural crest cells migrating from the neural tube into developing embryonic facial prominences. These neural crest cells interact with the developing brain via both physical contact and genetic signaling. Previous research has shown that the expression of Sonic hedgehog (*SHH*), fibroblast growth factor 8 (*FGF8*) and bone morphogenetic proteins (*BMPs *outline developmental interactions between the face and the brain in discrete temporal and regional patterns [18-22]. The connection of brain and facial phenotypes to their underlying genetic bases involves hierarchies of complex regulatory cascades, nested epigenetic networks and ever-changing patterns of cross-talk between molecules, cells and tissues throughout development [6,7,12,23]. Localization of facial phenotypic variations to specific areas of the face may reveal potential candidate genes and/or pathways targeted in the development of the brain in autism.

Given the clear evidence that the embryological face and anterior brain emerge and develop in exquisite intimacy [16,17], facial phenotypes can serve as accessible and informative indices of brain phenotypes in neurodevelopmental disorders. We propose that altered expression of genes involved in the development of neural tube structures and overlying facial prominences may result in distinct facial and neural phenotypes in ASD. We hypothesize that there are common autism-causing genes that affect early brain development and simultaneously the facial phenotype. Defining one or more common facial phenotypes within ASD will provide a new physical biomarker that can be used to improve ASD diagnoses, with all the associated benefits related to prognosis, recurrence counseling, choice of subgroup-appropriate therapies and the possibility of developing a screening tool to assist in early diagnosis.

Precise measures of phenotypes and innovative methods of analysis are integral to discovering the nature of developmental contributions to phenotypic variation. Using state-of-the-art three-dimensional photography to obtain facial images to precisely measure facial phenotypes, we tested two hypotheses. First, children with ASD display a subtle but distinct facial phenotype compared to typically developing (TD) children. Second, there are subgroups of facial phenotypes within the population of children with ASD that denote biologically discrete subgroups.

Previous work has suggested that there are autism facial phenotypes with a developmental basis. In an epidemiological study of facial photographs of children with autism and developmental disabilities, Rodier and colleagues [24,25] reported a facial phenotype common in autism consisting of decreased interpupillary distance (although intercanthic distance was not decreased), ptosis, strabismus, lop ears and hypotonia of the lower face. They postulated that these minor anomalies arise as the face is closing and the cranial nerves are invading mesenchyme that will develop into the muscle, skeletal and dermal tissues of the head. Rodier and colleagues [26,27] also suggested that facial phenotype might allow researchers to pick out the children whose autism is due to mutations in the homeobox genes which control the development of both the brainstem and face. Though this group of genes is important for embryological development, we now know that the face and the rostral brain are not patterned by genes in the homeobox family [28]. Additionally, Hammond *et al*. [29] studied a group of boys, ages 2 to 18 years from families with at least two affected family members, finding minor shape differences in comparisons of the mean facial phenotype of these boys to a control mean facial phenotype, with their major findings emphasizing significant facial asymmetry in boys with ASD and their family members and suggesting a shared developmental basis for these phenotypes.

**Figure 1:**
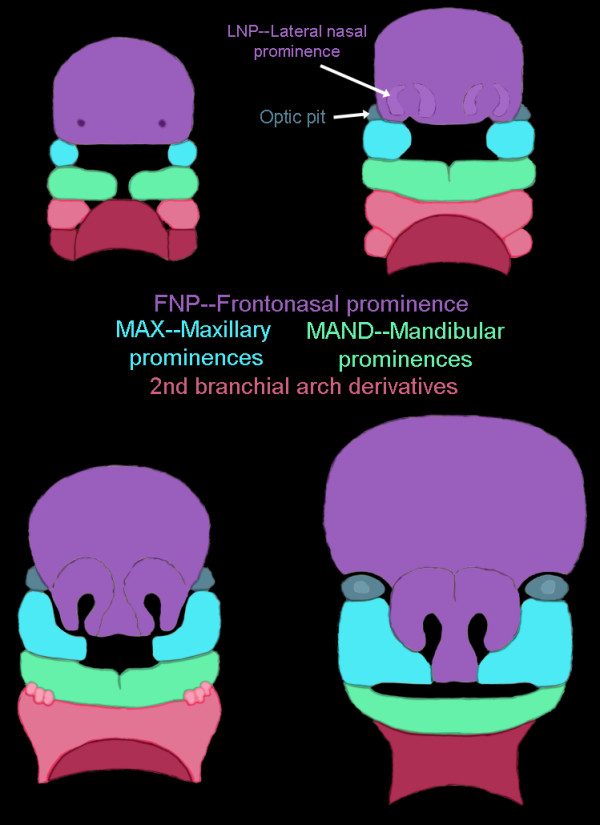
**Illustration of the seven facial prominences that give rise to specific regions of the face**. Frontonasal prominences (FNP) and lateral nasal prominences (LNP) are shown in purple, maxillary prominences (MAX) are shown in blue, mandibular prominences (MAND) are shown in green and second branchial arch derivatives are shown in shades of pink.

The embryonic face is derived from seven prominences that come together to form a face. These include the midline frontonasal process (FNP) and the paired lateral nasal prominences (LNPs) as well as maxillary prominences (MAXs) and mandibular prominences (MANDs) (Figure 1). The LNPs are very quickly assimilated into the FNP. Over the course of embryonic development, the facial mesenchyme of these developing prominences is bounded by the epithelia derived from both the forebrain neuroectoderm and the facial ectoderm [18]. The midline FNP forms from neural crest cell populations arising on the surface of the forebrain, migrating over the forebrain to become encased within the neural ectoderm of the forebrain and the facial ectoderm [19]. In fact, signals from the forebrain neuroectoderm are essential for the survival of the neural crest cells of the FNP [30], including SHH, FGF8 and BMP2 signaling [18-22]. This prominence gives rise to the forehead, the midline of the nose and the oral philtrum (Figure 1). LNP, MAX and MAND are also formed from neural crest and mesoderm cells, as well as from the epithelia of the facial surface ectoderm and pharyngeal endoderm [31]. These three laterally developing prominences are highly responsive to Wingless-type (WNT) signaling, whereas the midline FNP is not [32]. Similarly, regionalization of the neural tube is controlled by the *SHH*, *FGF *and *WNT *families [20]. *SHH *is expressed in the facial ectoderm and neuroectoderm at various developmental stages [31]. Thus face and brain in the embryo develop in concert, both temporally and genetically, and altered phenotypes of the face should reflect altered phenotypes of the brain via their shared developmental program.

## Methods

### Study sample and recruitment

A total of 105 boys ages 8 to 12 were included in the study (Table 1). The study was limited to boys to obviate any sex-related differences in facial phenotypes. The narrow 8- to 12-year-old age range was selected so that the boys were prepubertal but had completed 90% to 95% of head growth [33,34] and brain growth [35] and were at the same stage of facial development, which is a continuous process through the seventh decade of life [36]. To ensure that our sample populations did not differ in age, we employed a two-sample *t*-test using diagnosis (that is, ASD or not) as the categorical variable and age as the continuous variable. We determined that age was not significantly different between the two groups (*P *= 0.827).

**Table 1:** Study sample and age range

Study group	*n*	Age in years (mean ± SD)
Boys with ASD	64	10.14 ± 1.318
Typically developing boys	41	10.19 ± 1.261

ASD = autism spectrum disorder; SD = standard deviation.

Participants with ASD (*n *= 64) were recruited through the Thompson Center for Autism and Neurodevelopmental Disorders. All participants were screened before inclusion and met the following criteria: individuals were male; of Caucasian ethnicity; had not worn dental braces; were prepubertal (by parent report); were able to sit relatively still for picture-taking; had been diagnosed with Autistic Disorder, Asperger syndrome, or pervasive developmental disorder-not otherwise specified (PDD-NOS) according to the DSM-IV criteria prior to the day of the study; and had no additional syndrome diagnoses. Boys with fragile X syndrome and/or chromosomal disorders, including copy number variants (CNV), generalized dysmorphology or gestational age less than 35 weeks were excluded.

Of the 64 boys with ASD, 36 had completed the Simons Simplex Collection (SSC) protocol, which includes the Autism Diagnostic Interview, Revised (ADI-R) [37] and the Autism Diagnostic Observation Schedule (ADOS) [38], which were used in conjunction with the clinical judgment of one of the authors (JHM) to make the diagnosis of ASD. The 28 boys recruited through the Autism Medical Clinic were diagnosed on the basis of the DSM-IV criteria using a center-specific protocol based on the ADI-R together with the clinical judgment of the same author (JHM). The boys were assessed for generalized dysmorphology using the Autism Dysmorphology Measure [39].

TD boys (*n *= 41) were recruited from the Columbia, MO, USA, community via a notice published in the University of Missouri online information email and by word of mouth. Participants were screened using the same criteria described in the preceding paragraph, with the exception of a diagnosis of ASD. We chose TD boys as the control group, as our hypothesis was that development in ASD deviates from normal development. Samples were not matched for IQ, since this would not have allowed us to interpret how ASD deviates from the normal developmental trajectory. Recruitment and data collection procedures were carried out in accordance with approved Institutional Review Board protocols.

### Three-dimensional stereophotogrammetric imaging

**Figure 2:**
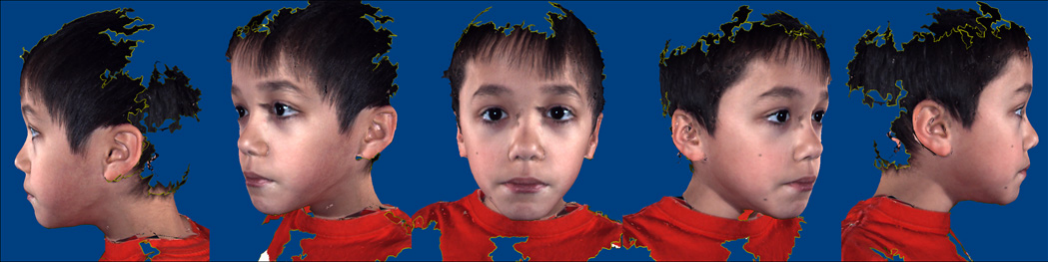
**3dMD image acquisition and analysis**. **(A) **Example of a 3dMD image acquired from an individual chosen at random from the study sample.

Three-dimensional images were acquired using the 3dMDcranial System (3dMD, Atlanta, GA, USA). Briefly, the 3dMDcranial System works by projecting random light patterns on the subject of interest (in our case, the human face). The subject is captured with multiple, precisely synchronized digital cameras configured in four modular units for a 360° full-head capture. Each unit contains three digital machine vision cameras. Because multiple cameras are used, there is no need for post-data capture ''stitching'' of multiple images into the single composite picture. Thus this technology removes a potential source of error by creating a valid three-dimensional representation of the subject at the time of data acquisition. Three-dimensional surface geometry and texture are acquired nearly simultaneously. Algorithms developed by 3dMD integrate the multiple images to produce a single three-dimensional image (Figure 2), which can be visualized and analyzed on a desktop computer using the 3dMD Patient software. A complete summary of the 3dMDcranial System is available online at http://3dMD.com/.

Prior to each session, all cameras were calibrated and tested to ensure that the images collected were consistent and usable. Individuals participating in the study were brought into the camera area and asked to sit as still as possible, look directly at one camera marked with a sticker and maintain a neutral expression (verbal instructions included closed mouth, no smile, no visible teeth and no raised eyebrows). Once the participant was comfortable and able to sit still, collection of the images began. Multiple pictures of each child were taken to ensure that the image used for analysis adequately captured all of the facial areas needed for landmarking.

### Anthropometric landmark data collection

**Figure 3:**
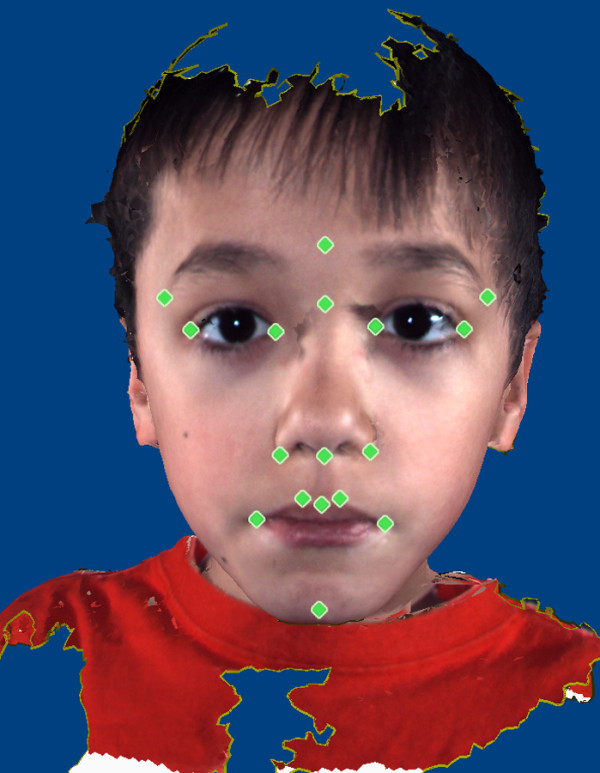
**Illustration of the anthropometric landmarks collected from the 3dMD images**. Landmarks are defined in Table 2.

Anthropometry, the biological science of measuring the size, weight and proportions of the human body [40], provides objective characterization of phenotypic variation and morphology. Facial anthropometry is performed on the basis of measures taken between landmarks defined on surface features of the face. The anthropometric landmarks defined by Farkas [40] located on the soft tissue of the face and head are repeatable, biologically relevant anatomical points. The three-dimensional landmark coordinate data were collected for 17 landmarks on the three-dimensional images by two raters (IDG and JRA) using the 3dMDpatient software program (Figure 3 and Table 2). Previous studies have shown three-dimensional landmark data collected from 3dMD images to be highly precise and repeatable [41,42]. All landmarks were checked for gross errors (for example, switching of right and left sides) prior to analysis.

**Table 2:** Three-dimensional anthropometric landmarks acquired from 3dMD images

Landmark names	Farkas abbreviations
Midline landmarks	
Glabella	g
Nasion	n
Subnasale	sn
Pogonion	pg
Bilateral landmarks	
Endocanthion	en
Exocanthion	ex
Alare	al
Crista philtre	cph
Chelion	ch
Labiale superius	ls
Frontotemporale	ft

Landmarks are illustrated in Figure 2, and their definitions can be found in Farkas [40].

To determine the reliability of data collection, an error study was performed. Four trials of landmark coordinate data were collected from two 3dMD images by both raters. Coordinate data were converted to all possible linear distances among the landmarks (all linear distances between 17 landmarks, resulting in 136 linear distances). Means, standard deviations and values of standard deviations as percentages of the linear distances were calculated for each linear distance in each observer's trials. The results derived by both raters are presented.

The ranges of the standard deviations expressed as percentages of linear distance were 0.19% to 8.11% for rater 1 and 0.19% to 14.3% for rater 2. Of the 136 total linear distances evaluated, linear distances with standard deviations greater than 5% of the mean totaled seven for rater 1 and eight for rater 2, leaving 129 and 128 linear distances, respectively, with less than a 5% error for each rater, respectively. Of the seven and eight linear distances with greater degrees of error, three of them were shared by the two raters.

The results of this error study indicate that the landmark coordinate data and the linear distances calculated from them can be collected with a very low degree of error. Therefore, the data collected by the two raters in this study are highly precise and repeatable.

### Morphometric data analysis

The landmark coordinate data collected using 3dMDpatient software were analyzed using Euclidean Distance Matrix Analysis (EDMA) [43], which is a linear distance-based morphometric method that does not rely on registration or fitting criteria [43,44]. Linear distances calculated between all possible pairs of landmarks were compared across samples as ratios. EDMA represents the form of each individual as a form matrix (FM), which is the set of all possible linear distances between the facial landmarks. Average FMs for each sample, that is, ASD and controls, are compared as ratios of like linear distances. This set of ratios of corresponding linear distances is called a "form difference matrix" (FDM). If a ratio in a FDM is equal to 1, then the faces being compared do not differ for that discrete linear distance. If the ratio is above 1, the linear distance is greater in the face used as the numerator. Likewise, if the ratio is less than 1, the linear distance is greater in the face used as the denominator. We used a nonparametric bootstrapping algorithm to calculate confidence intervals for each discrete linear distance to test for the significance of localized form differences [45]. The null hypothesis is that each discrete linear distance is similar for the two samples. Individual linear distances were considered significantly different if the calculated two-tailed 90% confidence interval did not include 1.0. Evaluation of confidence intervals for differences in specific linear distances enables localization of differences to specific facial regions.

This test of empirical differences in shape between samples is based on marginal confidence intervals of the bootstrap estimates of the linear distances between unique pairs of landmarks. Bonferroni-type corrections are not needed for these marginal confidence intervals, because in this approach multiple tests of linear distance differences using the same data are not conducted. Instead, with each bootstrapping step, all measures are estimated for an individual and tested in a high-dimensional space where each dimension represents a unique linear distance. The low-dimensional projection of these results for each linear distance is reported (see [43,45] for details).

This method has been used in numerous previous studies to compare facial morphologies (for example, see [42-49]) as well as the morphologies of other anatomic regions. A validation of this method for the data set in this study was performed to ensure that differences found in comparing the boys with ASD to TD boys using EDMA were not spurious. The group of TD boys was split into two randomly assigned age-equivalent groups. These two groups were then compared, and confidence intervals were calculated for each linear distance. On the basis of the results of these analyses, we determined that there were three significantly different linear distances among the total of 136 compared (2.2% of 136). These results show that fewer were statistically different than would be expected by chance (that is, 5%), demonstrating that this method is both sensitive and specific.

Principal Coordinates Analysis (PCOORD) application of EDMA was then performed on the scaled data for all participants in both groups [43-45,50]. This procedure is a form of clustering analysis that detects groups of forms with similar shapes and identifies linear distances that are influential in forming the defined clusters. In this procedure, the distribution of participants in multidimensional morphological space is examined. Unlike the form difference analyses described above, PCOORD compares individuals rather than samples. Axes are fitted through the shape space of this analysis such that the first axis accounts for the majority of the variation, the second axis accounts for the second-largest amount of variation and so on. These axes are referred to as the "first principal axis," the "second principal axis," and so forth. The position of participants along these axes is defined in terms of the linear distances between landmarks most highly correlated with these axes. Therefore, participants who cluster along a particular axis are similar in terms of the linear distances correlated with that axis. This analysis was performed to determine whether participants clustered on the basis of facial morphology, to identify the metrics that contributed to determination of the clusters and to explore the nature of the clusters to formulate hypotheses about the pattern of differences in the development of the face in children with ASD. The PCOORD analyses were performed on data that were scaled for differences in size. To do this, the FM for each individual was scaled such that each linear distance was divided by the geometric mean of all linear distances within that individual's FM. Thus each participant's data were scaled using a unique scaling factor. The geometric mean was chosen as a surrogate for size [51-53].

We analyzed all of the participants in the study to determine (1) whether there are aspects of facial morphology that distinguish the facial phenotypes of boys with ASD compared to TD boys and (2) whether there are facial subgroups within the ASD cohort that differ in their associated clinical and behavioral parameters.

### Behavioral and medical data

Each of the boys was evaluated for characteristics of their ASD diagnosis (social function, verbal function, repetitive behavior and language level), behavioral problems (aggression, attention deficits and self-injurious behaviors), outcome measures (IQ, communication, daily living skills, socialization and Vineland Adaptive Behavior Scale composite scores), the clinical course of their disorder (age at onset and presence of regression at onset), medical and neurological variables (seizures, electroencephalogram results, hypotonia, hypertonia, clumsiness, vision or hearing problems, tics, enuresis, handedness, feeding difficulties in infancy and allergies), physical morphology (head circumference, height, weight and dysmorphology) and family history of autism and related neuropsychiatric disorders among first-degree relatives.

The tests administered to all or the majority of participants included the ADI-R [37], ADOS [38], Social Communication Questionnaire (SCQ) [54], Vineland Adaptive Behavior Scale II [55], Peabody Picture Vocabulary Test (PPVT) [56], Child Behavior Checklist (CBCL) [57], an age- and development-appropriate IQ test (Full Scale IQ (FSIQ), Verbal IQ (VIQ), Nonverbal IQ (NVIQ)) and the Autism Dysmorphology Measure [39]. Not all measures of IQ were available for a small number of boys. NVIQ was available for the entire sample, FSIQ was available for all but one boy and VIQ was missing for four of the boys. Comprehensive prenatal, perinatal, teratogen exposure, development, general health, neurological and family histories (including income and education), were obtained using either the SSC Medical History or the Thompson Center Medical History, which record similar information. All participants received complete medical and neurological examinations, including assessment of growth and dysmorphology.

### Statistical comparisons of facial phenotypes with clinical and behavioral phenotypes

We compared clinical and behavioral traits to determine whether there were significant correlations between subgroup membership and the variables described in the preceding subsection. Continuous random variables were summarized by their mean, standard deviation and range. For categorical random variables, univariate comparisons of subgroup 1, subgroup 2 and the remainder were made using the χ^2 ^test or Fisher's exact test. For continuous variables, comparisons were made using Student's *t*-test. Because the IQ score is skewed, comparisons of IQ scores were made using the Kolmogorov-Smirnov test and the *t*-test after log-transforming IQ.

## Results

### Euclidean Distance Matrix Analysis

**Figure 4:**
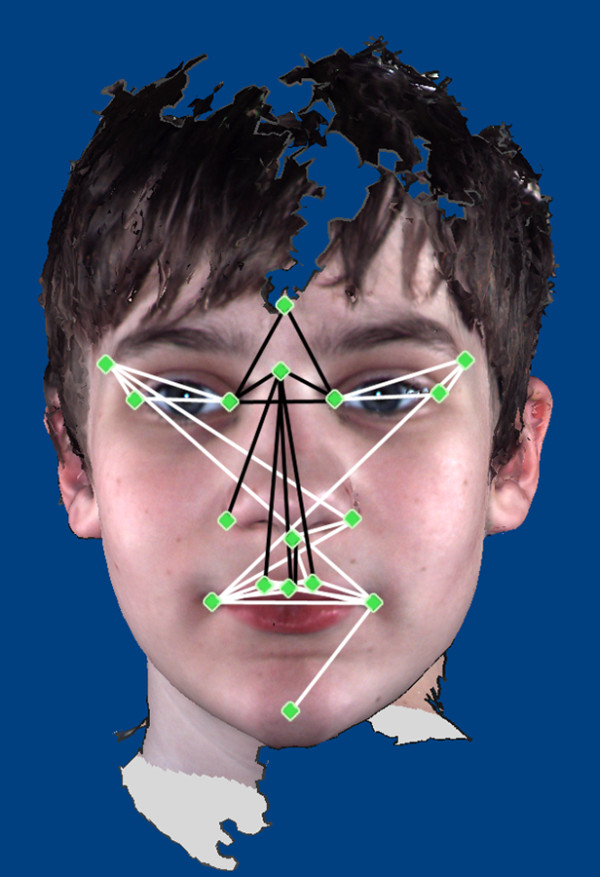
**Results of Euclidean Distance Matrix Analysis analyses of landmark coordinate data collected from 3dMD images**. White lines are statistically significantly increased in boys with autism spectrum disorder (ASD), and black lines are statistically significantly reduced in boys with ASD relative to typically developing (TD) boys.

Boys diagnosed with ASD demonstrate statistically significant differences in facial morphology compared to TD boys (Figure 4). Of the 136 total linear distances compared, 39 were statistically significantly different (28.7% of 136). Linear distances that were significantly reduced in the ASD group included those connecting glabella and nasion to the inner canthi and those connecting nasion with landmarks located on the nose and philtrum. Linear distances that were significantly increased in the ASD group connected the landmarks on the mouth with the inferior nasal region. Additionally, significantly increased linear distances connected the inner and outer canthi and the lateral upper face with the eyes and contralateral side of the mouth.

We did not find a statistically significant difference when we compared the face sizes of ASD and TD boys (*t*-test; *P *= 0.301). This suggests that the morphological differences found in the EDMA comparisons were not due to differences in size.

### Principal Coordinates Analysis

**Figure 5:**
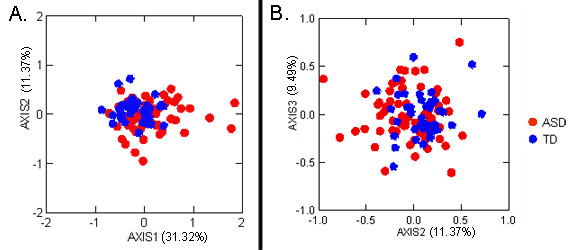
**Results of Principal Coordinates Analysis of landmark coordinate data collected from 3dMD images**. Red circles represent boys with autism spectrum disorder (ASD), and blue diamonds represent typically developing boys. **(A) **Plot of eigenscores for the first two principal axes. Axis 1 accounts for 31.09% of the variance within the entire sample, and axis 2 accounts for 11.33% of the variance. **(B) **Plot of eigenscores for the second and third principal axes. Axis 3 accounts for 9.44% of the sample variance.

The cluster analysis of all participants indicates that the majority of boys with ASD clustered with the TD boys. However, there are two subgroups of boys with ASD who were different from both the other boys with ASD and TD boys on the basis of overall facial morphology (Figure 5).

**Figure 6:**
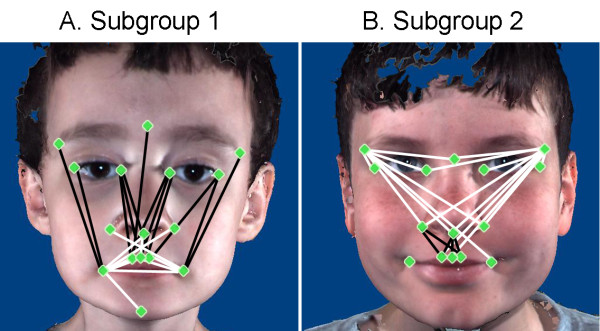
**Illustration of linear distances highly correlated with eigenscores on the first two principal axes of the principal coordinates analysis results**. **(A) **Subgroup 1 morphology. **(B) **Subgroup 2 morphology. Black lines are increased in boys in the subgroup, and white lines are reduced in boys in the subgroup.

The first principal axis accounted for 31.32% of the variance in the study population. Twelve of the individuals with ASD (18.8% of the ASD study group) clustered separately at the high positive end of this axis. The linear distances that were reduced in this subgroup relative to the remainder of the ASD sample population include those that connected landmarks at glabella, nasion and inner and outer canthi, with landmarks located on the mouth. The linear distances that were increased in this subgroup relative to the remainder of the ASD sample population include those spanning the breadth of the mouth and those connecting the corners of the mouth with the chin (Figure 6A).

The second principal axis accounted for 11.37% of the variance in the study population. Seven of the individuals with ASD (7.8% of the ASD study group) clustered at the strongly negative end of this axis. The linear distances that were reduced in this subgroup relative to the remainder of the ASD sample population spanned the area from the inferior aspect of the nasal region to the philtrum and lateral mouth. The linear distances that were increased in this subgroup relative to the remainder of the ASD sample population (1) spanned the breadth of the upper face and (2) connected the lateral aspect of the upper face with the inferior aspect of the nasal region (Figure 6B).

Correlations between axis score and age (*r*^2 ^= 0.125 for axis 1 and *r*^2 ^= 0.068 for axis 2) and between axis score and head size as measured by the geometric mean of all possible linear distances for each individual (*r*^2 ^= 0.285 for axis 1 and *r*^2 ^= 0.039 for axis 2) were low. These results suggest that group membership is not due to differences in age or head size.

### Comparisons of clinical and behavioral phenotypes among facial morphology subgroups

We found statistically significant correlations between subgroup membership and certain clinical and behavioral characteristics. Subgroup 1 (12 subjects) and subgroup 2 (5 subjects) were compared to each other and to the remainder (47 subjects). Comparisons that showed significant or nearly significant differences are given in Table 3. Subgroup 1 was characterized by increased autism severity scores on the SCQ, low frequency of Asperger syndrome diagnoses, lower cognitive scores based on significant FSIQ and NVIQ scores less than 70, increased regression, decreased macrocephaly and decreased total problem scores on the CBCL. By contrast, subgroup 2 was characterized by increased Asperger syndrome diagnoses, decreased autism severity scores on the SCQ and increased incidence of macrocephaly.

**Table 3:** Clinical and behavioral characteristics of autism spectrum disorder facial subgroups

Characteristics	Subgroup 1 *n *= 12 (18.8%)	*P *value	Subgroup 2 *n *= 5 (7.8%)	*P *value	Remainder *n *= 47 (73.4%)
Diagnosis					
Autistic disorder	↑75%	ns	↓20%	ns	62%
Asperger syndrome	↓8%	0.05*	↑60%	0.05*	32%
PDD-NOS	17%	ns	20%	ns	6%
Autism course					
Regression	↑58%	0.03*	↓20%	ns	25%
Autism severity					
SCQ lifetime, mean (SD)	↑25.3 (3.2)	0.03*, 0.05**	↓17.7 (9.2)	0.03***	20.5 (7.6)
SCQ <15	↓0%	ns	↑50%	ns	24%
IQ scores					
FSIQ, mean (SD)	78.4 (26.9)	ns	96 (36.2)	ns	87.9 (20.5)
FSIQ, range	36 to 113	ns	38 to 127	ns	31 to 130
FSIQ <70	50%	0.002**	↓20%	ns	15%
FSIQ ≥70	50%	ns	80%	ns	85%
VIQ, mean (SD)	78.4 (26.9)	ns	87.4 (39.2)	ns	87.5 (24.6)
VIQ, range	13 to 122	ns	23 to 121	ns	28 to 138
VIQ <70	42%	ns	20%	ns	24%
VIQ ≥70	58%	ns	80%	ns	76%
NVIQ, mean (SD)	79.2 (28.6)	ns	↑101.6 (31.0)	ns	90.3 (18.5)
NVIQ range	34 to 119	ns	53 to 121	ns	33 to 129
NVIQ <70	↑45%	0.02**	↑20%	ns	12%
NVIQ ≥70	55%	ns	↑80%	ns	88%
Language					
Mean age at first word month (SD)	15 (6.1)	ns	↓11.4 (4.3)	ns	18.6 (11.0)
Mean age at first phrase month (SD)	40 (31.5)	ns	23.2 (18.2)	ns	32.8 (16.5)
Behaviors					
CBCL total problems *t*-score ≥65	↓10%	0.01**	25%	ns	55%
Physical					
Macrocephaly	↓17%	0.02*	↑80%	0.02*, 0.02**	23%
Medical					
GI problems	33%	ns	↑80%	ns	36%
Neurological					
Seizures	33%	0.07**	0%	ns	11%
Infant feeding problems	8%	0.01**	↑40%	ns	51%
Family history					
SRS mother, mean (SD)	↑43 (21.4)	0.02**	25 (10.7)	ns	26.2 (20.4)

CBCL = Child Behavior Checklist; FSIQ = Full Scale IQ; GI = gastrointestinal; IQ = intelligence quotient; ns = not significant; NVIQ = Non-Verbal IQ; PDD-NOS = pervasive developmental disorder not otherwise specified; SCQ = Social Communication Questionnaire; SRS = Social Responsiveness Scale; VIQ = Verbal IQ. *Subgroups 1 and 2 comparisons. **Comparison of subgroups to the remainder.

No differences were found on the language measures, including the PPVT scores, Vineland Adaptive Behavior Scale II mean communication scores, ADI-R B language scores and the ADOS modules used. For all of the language measures, however, subgroup 2 subjects scored slightly better than subgroup 1. The age at first words and phrases illustrates the trend toward earlier language development in subgroup 2. No significant differences were found in the Vineland Adaptive Behavior Scale II scores, including Communication, Daily living skills, Socialization and Composite. No differences were seen for demographic or socioeconomic variables, including parent education or income. Since all subjects were male and 56% were also enrolled in the SSC, genetic indicators could be assessed. To be eligible for the SSC, there could be no history of autism among first- or second-degree relatives and no close relatives with major neuropsychiatric disorders. The only significant difference in the subgroups was a significantly higher Social Responsiveness Scale (SRS) mean score for mothers in subgroup 1.

## Discussion

We proposed that ASD might be associated with a distinctive facial phenotype and hypothesized that we might be able to identify subgroups within ASD based on facial phenotypes. Using sophisticated facial phenotyping based on three-dimensional stereophotogrammetric imaging and advanced statistical analyses, we developed a research methodology that allowed us to test and garner support for both hypotheses. In addition, we maximized our chances of success by selecting a relatively homogeneous ASD population that included only boys of self-reported Caucasian ethnicity, with essential autism, defined as having no discernible dysmorphology or microcephaly and within a limited age range.

We found that essential autism in boys is associated with a distinctive facial phenotype characterized by an increased breadth of the mouth, orbits and upper face, combined with a flattened nasal bridge and reduced height of the philtrum and maxillary region. This facial phenotype is similar to the one we recognized clinically (JHM) and may be the "beautiful face" mentioned by Kanner [58]. Embryologically, this facial phenotype is indicative of a perturbation of the FNP. It can be explained by reduction in the superoinferior dimension of the midline structures derived from the FNP, an increase in the subnasal portions of the FNP and a concomitant increase in the breadth of the upper face.

The common facial phenotype described by Rodier and colleagues [25] includes a reduced interpupillary distance with no difference in intercanthic distance. In contrast, in our present study, we found a narrowing of the intercanthic distance, or mild hypotelorism. These findings are complementary in that we found an overall decrease in intercanthic distance, which potentially translates to decreased interpupillary distance, although we did not directly measure that distance. Our findings are also in line with those of Hammond *et al*. [29]. However, the sample included in the Hammond *et al*. [29] study consisted of a group of boys, ages 2 to 18 years from families with at least two affected family members. Our study extends the findings of both Rodier and colleagues [25] and Hammond *et al*. [29] by quantifying precisely localized differences and variations in facial phenotypes in a homogeneous group of boys with essential autism.

Our findings further extend previous work in that we have discovered two subgroups of boys with ASD who displayed unique facial phenotypes, which correspond to distinct clinical phenotypes, compared to both the majority of boys with ASD and TD boys. Subgroup 1 displays decreased height of the facial midline and increased breadth of the mouth as well as the length and height of the chin. These regions of the face develop primarily from the FNP and midline portions of the MAND prominences of the embryonic face. Subgroup 2 displays increased breadth of the upper face in combination with decreased height of the philtrum. Both of these regions develop from the embryonic FNP.

The results of the tests of our hypotheses indicate that boys with ASD have an altered developmental pattern of the structures derived from the embryonic FNP and the MAND. It is well-documented that the developing FNP is derived from localized, specific cell populations under patterned genetic control. A number of developmental genes have been implicated in patterning the outgrowth of FNP, including *FGF8*, *SHH *and *BMP2*.

Neural crest cells that ultimately make up the FNP migrate over the forebrain to become encased within the neural ectoderm of the forebrain and the facial ectoderm [19]. *FGF8 *plays a chemoattractive role in neural crest cell migration [20]. Signals from the forebrain neuroectoderm are essential for the survival of the neural crest cells of the FNP [30], including SHH-dependent signaling from forebrain [21]. Other studies have shown that SHH provides a key signal in regulating facial neural crest cell survival and patterning [22] and regulates *BMP2 *expression in the middle and upper face [18]. Furthermore, it has been shown in an animal model that decreased SHH signaling leads to narrowing of the FNP and hypotelorism [59]. The MAXs and LNPs are responsive to WNT signaling [32]. WNT signaling leads to elevated cell proliferation and migration of neural crest cells [32]. No midline structures are responsive to WNT.

Correct patterning and development of the forebrain also requires a balance of these genetic signaling factors [18], including SHH and the FGF, BMP and WNT families [20,60]. *SHH *is expressed in the facial ectoderm and neuroectoderm at various developmental stages and acts synergistically with *FGF8 *in both face and brain development [31]. *SHH *expression in facial ectoderm affects the expression of *FGF8 *in the brain [30]. Likewise, expression of *FGF8 *by the forebrain is stimulated by the presence of neural crest cells [61].

It is clear that development of the face and brain is an interactive process, both anatomically and genetically. Gene expression studies have shown that facial and neural tube development are intimately interrelated. Altered gene expression patterns are associated with alterations in face and brain development. The reverse is also true: Altered face and brain development is associated with alterations in gene expression. We know that the brain is altered in people with ASD (reviewed in [62]), and the results of our present study show that the face is also affected. However, the sequence of events leading to these differences is unclear and may differ among the various subgroups described herein.

There is evidence derived from genetic studies implicating the developmental genes that control the patterning of the FNP and forebrain which causes autism. The *SHH *gene, though not identified by autism linkage or association studies, is functionally related to the *Patched *gene (*PTCHD1*), which is a strong autism candidate gene. Investigators who conducted CNV studies [63] first identified *PTCHD1 *gene microdeletions and missense mutations in males with ASD [64]. *SHH *signaling repression is relieved when SHH binds to PTCHD1. *BMP *is part of the functional face and brain patterning network interacting with *SHH and FGF8 *to maintain brain and face patterning. Bakrania *et al*. [65] evaluated gene expression in embryos and demonstrated cotemporal and cospatial expression of *BMP4 *and *SHH *signaling genes. It is expected that sophisticated brain and/or face functional transcriptome studies may be useful in further linking genes involved in simultaneous face and brain development.

The clinical and behavioral differences that we identified between subgroup 1 and subgroup 2 boys support our hypothesis that the subgroups are biologically and etiologically distinctive. Subgroup 1 appears to be more severely autistic, with only 8% diagnosed with Asperger syndrome compared to 60% in subgroup 2, and has higher SCQ lifetime scores than subgroup 2 (25.3 vs 17.7). The percentage of IQ scores less than 70 were higher in subgroup 1 than in the other groups: 50% on the FSIQ and 45% on the NVIQ, compared to only 20% on both the FSIQ and NVIQ (one of five) in subgroup 2. and 15% FSIQ and 12% NVIQ in the ASD remainder group. Verbal IQ scores were also lower in subgroup 1 but did not reach statistical significance. Subgroup 1 also displayed several features predictive of poor outcome, including a higher risk for seizures and increased incidence of language regression at ASD onset. The observation of significantly higher SRS scores in mothers of boys in subgroup 1 is interesting but unexplained at this time.

By contrast, subgroup 2 appears to be aligned more with an Asperger syndrome diagnosis, which was made in 60% of subgroup 2 participants compared to only 8% in subgroup 1 and 32% in the remainder. Lifetime SCQ scores in subgroup 2 were also lower (17.7) than those in subgroup 1 (25.9) and the remainder (20.6). Consistent with the Asperger syndrome diagnosis, boys in subgroup 2 spoke their first words significantly earlier than boys in subgroup 1 and the remainder, and they were significantly more likely to be macrocephalic (80% vs 17%) compared to subgroup 1. Though IQ score differences did not reach significance within this small subgroup, boys in subgroup 2 had consistently higher FSIQ, NVIQ and VIQ scores than boys in subgroup 1.

Though we maximized our study outcomes by using a relatively homogeneous group of boys of Caucasian ancestry with essential autism within a narrow age range, the subject group was imperfect in a number of ways. Though 56% of the boys had participated in the SSC, not every participant completed the entire test battery, which slightly decreased the number of subjects who could be analyzed statistically for some comparisons. However, all of the four boys without VIQ scores and the one boy without a FSIQ score clustered morphologically within the main group and not within either subgroup. Thus our findings are highly unlikely to be affected by the small number of missing data points. In addition, the SSC population is biased toward a higher-functioning group of boys, which tended to shift the cognitive and outcome curves. The higher-functioning population recruited from the SSC may, however, have helped delineate subgroup 2 by increasing the proportion of subjects with Asperger syndrome. In addition, the number of boys in each subgroup was small. One additional observation is that in subgroup 2, which contained only five boys, one of the five was an outlier with significantly lower scores on IQ, language and outcome measures. This suggests that in future studies of larger numbers of subjects, we may find that subgroup 2 will be dissected into subgroups 2A and 2B. Finally, although one of the strengths of our study is that it comprised a homogeneous group of boys, that is, narrow age range, single sex, limited ethnic diversity and diagnosis made by a single clinician, it remains to be seen whether our findings will be consistent in a more heterogeneous population.

## Conclusions

Differences in facial morphology may reflect alterations in embryologic brain development in children with ASD. Our results suggest potential differences in etiologies for the various subgroups of children. Further investigations into brain morphology will help to elucidate the causes and significance of these subtle differences. Verification of the role of a number of neurodevelopmental candidate genes may also be expedited by restricting analyses to the more homogeneous autism subgroups described herein. Likewise, based on our understanding of facial and neural development, identification of specific neurodevelopmental genes responsible for autism suggests which regions of the embryonic brain are most apt to be affected, providing potential target structures for future investigation.

## Consent

Written informed consent was obtained from the parents of the patients for publication of this research article and any accompanying images. A copy of the written consent is available for review by the Editor-in-Chief of this journal.

## Competing interests

The authors declare that they have no competing interests.

## Authors' contributions

KA supervised 3dMD data collection, analyzed 3dMD anthropometric data, interpreted results of analyses, prepared manuscript figures, contributed to the conception and design of the study and is the primary author of the manuscript. IDG collected 3dMD photographs, collected 3dMD anthropometric data and maintained the image data archive. KKC collected 3dMD photos, recruited subjects and prepared the figures. JRA collected 3dMD anthropometric data and maintained the image data archive. TNT recruited subjects, maintained the behavioral and clinical data archive, analyzed behavioral and clinical data and participated in drafting and revising the manuscript. YD supervised recruitment of the control sample and contributed to the conception and design of the study. JHM collected the behavioral and clinical data, supervised analyses of these data, contributed to the conception and design of the study and contributed to drafting and revising the manuscript. All authors read and approved the final manuscript.
